# Unexplained Early Infantile Epileptic Encephalopathy in Han Chinese Children: Next-Generation Sequencing and Phenotype Enriching

**DOI:** 10.1038/srep46227

**Published:** 2017-04-07

**Authors:** Ahmed Arafat, Peng Jing, Yuping Ma, Miao Pu, Gai Nan, He Fang, Chen Chen, Yin Fei

**Affiliations:** 1 Xiang Ya First Hospital of Central South University, China, 87 Xiang ya road, Changsha, Hunan, 410008, P.R. China

## Abstract

Early Infantile Epileptic Encephalopathy (EIEE) presents shortly after birth with frequent, severe seizures and progressive disturbance of cerebral function. This study was to investigate a cohort of Chinese children with unexplained EIEE, infants with previous genetic diagnoses, causative brain malformations, or inborn errors of metabolism were excluded. We used targeted next-generation sequencing to identify potential pathogenic variants of 308 genes in 68 Han Chinese patients with unexplained EIEE. A filter process was performed to prioritize rare variants of potential functional significance. In all cases where parental testing was accessible, Sanger sequencing confirmed the variants and determined the parental origin. In 15% of patients (*n* = 10/68), we identified nine *de novo* pathogenic variants, and one assumed *de novo* pathogenic variant in the following genes: *CDKL5* (n = 2), *STXBP1* (n = 2), *SCN1A* (n = 3), *KCNQ2* (n = 2), *SCN8A* (n = 1), four of the variants are novel variants. In 4% patients (*n* = 3/68), we identified three likely pathogenic variants; two assumed *de novo* and one X-linked in the following genes: *SCN1A* (n = 2) and *ARX* (n = 1), two of these variants are novel. Variants were assumed *de novo* when parental testing was not available. Our findings were first reported in Han Chinese patients with unexplained EIEE, enriching the EIEE mutation spectrum bank.

Epilepsy is one of the most common neurologic disorders, with a prevalence of 5–10 per 1,000/year^1^. Early infantile epileptic encephalopathies (EIEEs) are a heterogeneous group of disorders characterized by intractable seizures and unremitting interictal paroxysmal epileptiform activity that consequently impair neurodevelopmental outcomes during the first year of life^2^,^3^. It is one of the most severe and earliest form of epilepsy^4^. Genetic causes should be considered in the absence of structural brain abnormalities or inborn errors of metabolism^5^. A genetic cause for an epileptic encephalopathy was first recognized in 2001, when all seven children who were recruited in a study of Dravet syndrome had a de-novo *SCN1A* mutation^6^. Now molecular techniques, such as chromosomal microarray and next generation sequencing (NGS) of multiple genes, have contributed to today’s rapid growth in gene discovery for epileptic encephalopathies^7^,^8^,^9^, Copy number variants (CNVs) are important molecular causes of epileptic encephalopathy, with up to 8% of cases showing a causative or potentially contributing CNVs^10^.

As molecular diagnostics evolve, and with the ease of using them in some advanced facilities besides the underlying burdens of epilepsy especially in infancy, there is always a need to demonstrate the various clinical and research approaches. Profound understanding of the broader clinical spectrum and interpretation of genotype correlations requires accurate phenotyping. In this study we describe a cohort of previously investigated infants with unexplained sporadic EIEE and report the use of targeted NGS, followed by analysis of selected epilepsy genes in the probands.

## Methods

### Patients

Sixty eight infants with EIEEs were recruited for our study from Department of Pediatrics Neurology, Central South University, Xiangya first hospital, from 2012 to 2015 by a pediatric neurologist. Standardized clinical information was collected using a pre-test questionnaire completed by the recruiting clinician. Lymphocyte DNA was collected in all cases and their parents (as possible as paternal testing was accessible) using standard procedures. All clinical, neurophysiology, and imaging data were checked carefully to clearly define phenotypes. Phenotypes were classified into known electroclinical syndromes according to International League Against Epilepsy (ILAE) classification, where possible, or the electroclinical evolution defined as “unclassified”.

All patients were enrolled according to the following criteria; seizures onset within a year of age; severe electroencephalography EEG findings; Intellectual disabilities (ID); and no pinpointed cause. Patients who were examined previously for inborn errors of metabolism, structural brain malformation and common causes of EIEEs were excluded by detailed histories and routine examinations including blood glucose, blood ammonia, lactic acid, serum electrolytes, cranial magnetic resonance imaging (MRI), brain Computed Tomography (CT), amino acid and organic analyses, urinary metabolic screening, chromosome karyotype analysis and copy number variations. In addition, patients with monogenic disorders such as tuberous sclerosis complex, typical Rett syndrome, and MMPSI with *KCNT1* ([Online Mendelian Inheritance in Man, http://www.omim.org/] OMIM: 608167) variants were not enrolled in our cohort. All patients were ethnically Han Chinese. Our study included 44 cases of West syndrome, four cases of Dravet syndrome, two cases of Ohtahara syndrome and eighteen cases were unclassified EIEEs. Clinical information, including clinical manifestation, EEG, MRI, CT and family history were collected and patients were followed up either by phone calls or inpatient/outpatient visits.

To assess intellectual disabilities in our patients we used the diagnostic criteria of the *DSM-5* for Intellectual disabilities (*Diagnostic and Statistical Manual of Mental Disorders, Fifth Edition, American Psychiatric Association, 2013*), we assessed our patients adaptive functioning through observations at home and school, clinical interviews and standardized age-related rating scales as follow; for patients younger than 2–4 years of age we used Gesell Developmental Schedules, for patients between 4–6 years we used the Wechsler Preschool and Primary Scales of Intelligence Fourth Edition (WPPSI-IV), and for patients who are 6 years old or older 6 we used the Wechsler Intelligence Scale for Children, Fourth Edition (WISC-IV). Patients with deficits in their adaptive and intellectual functioning with an onset during their developmental period were classified into mild ID when their development quotient (DQ) or intelligence quotient (IQ) values were between 50 to 69, moderate ID when DQ/IQ values were between 35 to 49 and severe ID when DQ/IQ values were less than 35.

Written informed consent was obtained from each patient and their participating parents. Methods were carried out in accordance with the relevant guidelines and regulations. This study was approved by the Central South University First Hospital Medical Ethics Committee.

### Targeted Next-Generation Sequencing

We selected 308 genes for analysis in the panel including 16 known epilepsy-associated genes; genes analyzed were *ARX, CDKL5, SLC25A22, STXBP1, SPTAN1, SCN1A, KCNQ2, ARHGEF9, PNKP, SCN2A, PLCB1, SCN8A, KCNT1, TBC1D24, GABRA1* and *SYNGAP1* (see Supplementary Table S1). A custom-designed panel capturing the Exon regions of 308 genes associated with early infantile epileptic encephalopathy was synthetized using the Agilent Sure-Select Target Enrichment technique. Targeted next generation sequencing (NGS) was subsequently performed on an Illumina Hiseq 2000 platform (Illumina, San Diego, CA, USA) using a paired-end sequencing of 100 bp to screen for variants. Multiple sequence alignments of the affected amino acids were performed using a sequence alignment (Clustal W; The Biology Workbench, San Diego, CA, U.S.A.). Image analysis and base calling were performed by RTA software (real-time analysis, Illumina) and CASAVA software v1.8.2 (Illumina). After marking duplicate reads and filtering out reads of low base quality score using the Genome Analysis Tool kit (GATK), Sequence reads in FASTQ format were aligned to the reference human genome (hg19) using BWA (0.6.1-r104) and default settings, using BWA software (Pittsburgh Supercomputing Center, Pittsburgh, PA, USA)^11^. In addition to insertion-deletions (indels) and single-nucleotide polymorphisms (SNPs) identified using the GATK, variants were annotated using ANNOVAR (www.openbioinformatics.org/annovar/annovar_download.html#credit). The average sequencing depth was 140×.

**Figure 1:**
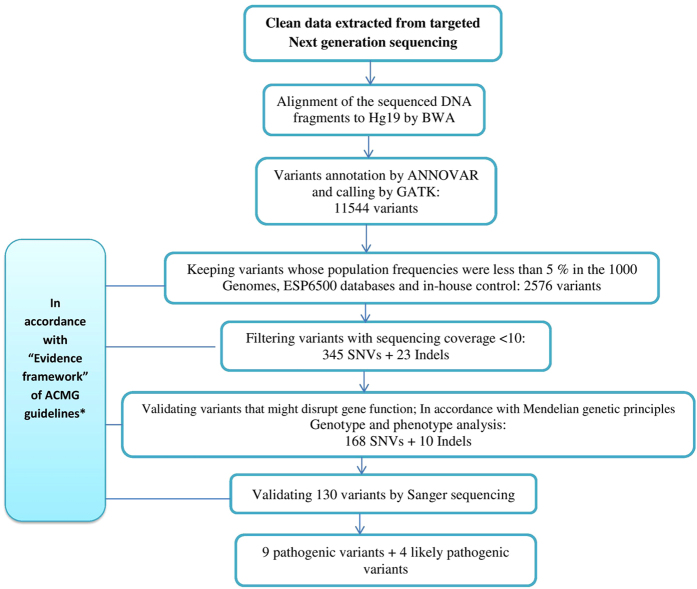
Screening of Potentially Pathogenic and Likely Pathogenic Variants in Our Study of 68 Patients with Unexplained EIEE. *Richards, S. *et al*. Standards and guidelines for the interpretation of sequence variants: a joint consensus recommendation of the American College of Medical Genetics and Genomics and the Association for Molecular Pathology. *Genet. Med.* **17**, 405–423 (2015)^12^.

In accordance with ACMG Standards and Guidelines^12^, we performed several steps of filtering data to identify possible pathogenic variants: (a) Obtaining the frequencies of variants in population databases; Exom Aggregation Consortium, 1000 Genomes Project and ESP6500 databases and in-house control (200 healthy controls were used by the company which performed NGS for our team); (b) Assessment of variants pathogenicity in disease databases; OMIM, Human Gene Mutation database and ClinVar; (c) Determination of the effect of the variant on the primary and alternative gene transcripts, other genomic elements, as well as the potential impact of the variant on the protein through computational (in silico) predictive programs, PlyPhen-2 and SIFT. Variants validated after the above noted steps were then checked in the published literature where possible and considered to be a candidate for pathogenic variants and were picked out for further investigation. In accordance with Mendelian genetic principles (the inheritance pattern of the involved genes) we chose variations which to validate by Sanger sequencing to identify the segregate status in these families and indicate the candidate pathogenic variants according to parental origin of the variations and clinical features of the patients. Candidate pathogenic variants were then assessed in accordance with ACMG standards and guidelines’ “Evidence framework’ to be classified into pathogenic and likely pathogenic variants (see Fig. 1).

## Results

### Clinical Characteristics

We recruited 68 infants with unexplained early infantile epileptic encephalopathy, all were less than a year old; male to female ratio was 1:0.45. We had 44 cases of West syndrome (n = 44), four cases of Dravet syndrome (n = 4), two cases of Ohtahara syndrome (n = 2) and eighteen cases were unclassified EIEEs (n = 18). Seizures onset was as follow; 24 cases within three months of life, 29 cases from three to six months of life and 15 cases from seven to twelve months of life, average age of seizures onset was 4.65 ± 2.37 months, thus 78% (n = 53) of cases developed epileptic encephalopathy within six months of life. Assessment of patients’ intellectual disability revealed 18 patients with mild ID (26.4%), 20 patients with moderate ID (29.4%) and 30 patients with severe ID (44.2%). In our patients, family history was positive in nine patients where their first degree relatives have had epilepsy or intellectual disability and mother’s pregnancy history also was positive in nine patients. Table 1.

In West syndrome cases (n = 44), seizures onset was as follow; 12 cases before three month of life, 22 cases from three to six months and ten cases from seven to twelve months of life, the seizures types were as follow; 31 patients had infantile spasms, 4 had spasms and tonic seizures, 5 had tonic-clonic seizures, and 4 with partial, tonic or tonic-clonic seizures. Their EEG showed Hypsarrhythmia including five patients with 50% of their epileptiform discharges happened during the non-rapid eye movement sleep cycle. Assessment of their ID showed 12 patients with mild ID, 10 patients with moderate ID and 22 patients with severe ID.

As noted above, the other cases of unexplained EIEE (n = 24) included four cases of Dravet syndrome, two cases of Ohtahara syndrome and eighteen cases were unclassified EIEEs (n = 18), in these cases seizures onset was as follow: 12 cases before three month of life, 7 cases from three to six months and 5 cases from seven to twelve months; the seizures types were as follow: 2 patients had infantile spasms, 7 had tonic-clonic seizures, 9 with partial seizure, 1 with tonic seizure and 5 had multiple types of seizures. EEG findings in these cases are described as follow: intermittent burst-suppression during sleep cycle in four cases; spikes, sharp waves and polyspikes in four cases; spikes, slow-spike-and-wave and polyspike-and-slow-wave complexes in five patients; intermittent hypsarrhythmia in four cases; sharp waves in three cases; slow waves with high amplitude in two cases; widely spread slow-spike-and-wave complexes in one case with 100% of these epileptiform discharge happened during the non-rapid eye movement sleep cycle and one case of slow basic background activity rhythms. Assessment of ID in these cases showed 6 patients with mild ID, 10 patients with moderate ID and 8 patients with severe ID.

Among the 68 patients recruited in our cohort, the efficacy of antiepileptic drugs (AEDs) was illustrated as follow; clinical seizure freedom was achieved in 13 patients, 16 patients had their seizures controlled, 28 patients were resistant to treatment, three patients have died (one case probably due to nocturnal asphyxia, one case of probable sudden unexpected death in epilepsy (SUDEP) and one case of unknown cause of death). We lost contact with eight patients. Patients’ response to AEDs had been followed up from one month to four years. Patients were treated with a single or poly AEDs, adrenocorticotropic hormone (ACTH) and/or ketogenic diet. AEDs were chosen according to patients’ response to treatment. AEDs options were; Oxcarbazepine (OXC), Carbamazepine (CBZ), Levetiracetam (LEV), Phenobarbital (PB), Topiramate (TPM) and Sodium valproate (VPA). Clinical features of the 68 patients in our cohort including MRI and CT scan findings are summarized in Table 1.

### Identification of Variants

Of the 68 patients with unexplained EIEEs, variants were detected in 13 patients (19%). Nine *de novo* pathogenic variants including four novel variants, and one assumed *de novo* pathogenic variant were identified in 15% of patients (*n* = 10/68). Variants in these patients and associated phenotypes are described as follow: two variants of *CDKL5* (c.278dupA/p.E93fs, c.1110delC/p.N370fs) and one variant of *STXBP1* (c.1216C > T/p.R406C) were identified in three West syndrome patients; Three variants of *SCN1A* (c.225G > T/p.E75D, c.2134C > T/p.R712X, c.4811G > A/p.W1604X) were associated with three Dravet syndrome patients; two *KCNQ2* variants (c.1574G > A/p.R525Q, c.1574G > A/p.R525Q) and one *SCN8A* variant (c.5615G > A/p.R1872Q) were identified in three patients with unclassified EIEEs; one *STXBP1* variant (c.1216C > T/p.R406C) was identified in a patient with Ohtahara syndrome.

**Figure 2:**
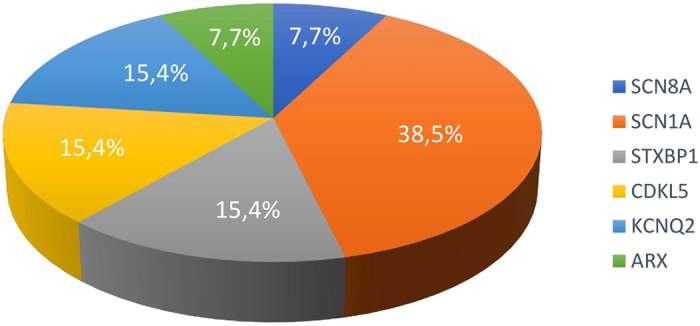
Percentage of Cases with Variants Among All the Variants Identified in Our Study. Among the 13 cases with detected variants, *SCN1A* was the most frequently affected gene in our study, accounting for 38.5%, followed by *STXBP1, CDKL5, KCNQ2, ARX and SCN8A* of 15.4%, 15.4%, 15.4%, 7.7% and 7.7% respectively.

Three likely pathogenic variants; two assumed *de novo* and one X-linked were identified in 4% patients (*n* = 3/68) in the following genes: *SCN1A* (n = 2) and *ARX* (n = 1), two of these variants are novel. Variants in these patients and associated phenotypes are described as follow; two variants of *SCN1A* (c.1703G > A/p.R568Q, c.4176T > A/p.N1392K) were identified in two patients of unclassified EEES and one variant of *ARX* (c.1600G > C/p.A534P) in a West syndrome patient. Variants were assumed *de novo* when parental testing was not available. In accordance with ACMG Standards and Guidelines and its rules for combining criteria to classify sequence variants, variants were classified into pathogenic and likely pathogenic variants^12^. Among the 13 cases with detected variants, *SCN1A* was the most frequently affected gene in our study, accounting for 38.5% (5/13), followed by *STXBP1, CDKL5, KCNQ2, ARX and SCN8A* of 15.4% (2/13), 15.4% (2/13), 15.4% (2/13), 7.7% (1/13) and 7.7% (1/13) respectively (see Fig. 2). Phenotypes, inheritance, and molecular characteristics for all patients with variants are described in Table 2.

## Discussion

Wide range of genotype and phenotype heterogeneity makes it difficult to predict with certainty the potentially responsible gene for many EIEEs. Our group has conducted this study on Chinese Han infants. In our study the total detection rate of variants was 19% (13/68) including pathogenic variants in 15% of the cases (10/68), and likely pathogenic variants in 4% of the cases (3/68). Variants were found in patients with a broad range of phenotypes (see Table 2). Perinatal Sodium channelopathies were identified in six patients with variants in *SCN1A* and *SCN8A*, five and one respectively.

*De novo* variants in *SCN1A* are an increasingly recognized cause of an early-onset seizure and developmental delay. Roughly 80% of Dravet syndrome patients carry a mutation in the *SCN1A* gene^13^,^14^,^15^. Variants in *SCN1A* identified in our study are described as follow: Three *de novo* pathogenic variants of *SCN1A* including two novel variants were detected in three cases of Dravet syndrome (C0125, C0129 and R1014), several studies have supported the association of *SCN1A* gene mutation and Dravet syndrome^13^,^14^,^15^. In case (C0125), no remarkable ID was noticed till the age of one year, at the age of two years old she could walk and talk, at the age of four years she would face some difficulties climbing up and down stairs, her DQ at the age of four years and two months indicates moderate ID. (C0129) was delivered at the age of 32 weeks. Till the age of two years no remarkable ID was noticed and at the age of three years and five years his DQ indicates mild ID. (3). In case (R1014) no remarkable ID was noticed before the age of two years. but then he started to lag behind his peers and his DQ indicates moderate ID at the age of 2 years and six months. For the above mentioned cases parental testing was available. Two likely pathogenic variants of *SCN1A* including one novel variant were detected in two unclassified EEEs (C0117, S560) parental testing was not possible. (C0117) was delivered at the age of 28 weeks and was diagnosed with hyperbilirubinemia, at the age of one year and one month his DQ indicates moderate ID. (2). (S560) female, at the age of two years and four months her DQ indicates moderate ID. The variant we identified in (C0125) was previously reported and led to protein change (Arg712X)^16^. Although these observations provide further support for the proposed association of *SCN1A* variants and Dravet syndrome^17^,^18^, further studies are needed to confirm the linkage between *SCN1A* variants and other unclassified EEEs. It worth mentioning that most of reported *SCN1A* variants in patients with seizures onset within the first year of life were associated with severe developmental delay, while in our study, *SCN1A* carrying cases were shown mild to moderate ID, this finding could be due to the small size of our cohort or population diversity and different inclusion criteria. *De novo* variants in *SCN8A* are a recently recognized cause of early-onset seizures with moderate to severe developmental delay^18^,^19^,^20^,^21^,^22^,^23^. We identified in one case (S557) with unclassified EEE, two *de novo* novel variants; *SCN8A* (NM_014191) c.5615G > A/p.R1872Q and *KCNMA1* (NM_001014797) c.3488A > G/p.N1163S. This case was a male patient with irrelevant perinatal and family histories; now at the age of two, he suffers severe epilepsy, he cannot speak or walk, and his DQ shows moderate ID. Previous studies have proposed that *de novo* variants may often be pathogenic variants in a child with severe epilepsy and negative family history^21^. *De novo SCN8A* heterozygous variants also have been proven to be pathogenic^18^,^19^,^20^,^21^,^22^, especially in patients with seizures onset within the first year of life^23^, which matches our findings. To our knowledge, there is only two reported cases of *KCNMA1* variants; first study, Tomas M *et al*.^24^, when he reported a relation between *KCNMA1* and severe essential hypertension and myocardial infarction^24^, second, Du W *et al*.^25^, when they reported that *KCNMA1* mutation would result in generalized epilepsy and paroxysmal dyskinesia^25^. We are here the first to report the possible association between *KCNMA1* gene variant and unclassified EEEs. We did not discuss this finding in details for the possibility that the phenotype of the patient was mainly due to the pathogenic variant of *SCN8A.*

Heterozygous variants in *KCNQ2* are a well-understood cause of early-onset seizures. Reported phenotypes differ from benign familial neonatal seizures to a progressive pharmacoresistant EIEE^26^,^27^. We identified two *KCNQ2* variants, in two cases of EIEE (S559, C0107), both carried the same mutation in *KCNQ2* gene: NM_004518, c.1574G > A/p.R525Q. The variant in the first case (S559) was *de novo*, while for the other one (C0107) parental testing was not possible. (S559) had refractory seizures till the age of a year and five months, after she was given monotherapy of LEV, she was declared seizure-free. Mild ID was detected as early as four months of age, now at the age of four years her DQ indicates mild ID. (2). (C0107) had refractory seizures till she was given VPA and LEV at the age of one year and one month. Now at the age of one year and six months seizures are controlled and her DQ indicates moderate ID. Although several studies have reported that variants with *KCNQ2* are usually associated with severe developmental delay^17^,^28^, our cases showed mild and moderate ID, we refer this to early control of seizures and the efficacy of AEDs. A recent study has reported the efficacy of VPA, LEV and TPA with patients who carry *KCNQ2* variants^28^. Formerly reported genotype-phenotype studies have emphasized that truncating variants of *KCNQ2* are associated with benign, inherited phenotype (benign familial neonatal seizures1)^26^, while missense variants of *KCNQ2* are the causative variants of severe, sporadic phenotypes^27^,^29^ (EIEE), cellular experiments point out that these last-mentioned variations may have a dominant negative effect on the function at a cellular level^27^,^29^, thus our findings are adding momentum to the fact that The *KCNQ2* gene, is responsible for about 10% of EIEEs with neonatal onset^30^.

*De novo* missense variant in *STXBP1* was identified in two cases (C0108, R1007), the seizures in both cases were refractory. (C0108) was diagnosed with West syndrome, now at the age of three years and ten months he cannot sit alone or call a person and his DQ indicates severe ID. (R1007) was diagnosed with Ohtahara syndrome, now at the age of one year old his DQ indicates moderate ID. Both of these patients carried the same missense variant in *STXBP1* gene: (NM_003165), c.1216C > T; p.R406C, this variant was reported as a pathogenic variant in a case of Ohtahara syndrome with profound ID^31^, which matches our result and support the findings of previously reported studies that mutation in *STXBP1* is extensively associated with severe early-onset epileptic encephalopathies including Ohtahara syndrome, West syndrome and other epileptic phenotypes with moderate to severe ID^32^,^33^,^34^,^35^.

An *ARX* gene variant was identified in our study in a male case (S569) with West syndrome; it revealed maternally inherited in the *ARX* gene (NM_139058, c.1600G > C/p.A534P) of a non-consanguineous marriage and irrelevant perinatal and family histories, it was deemed as a likely pathogenic variant. Seizures were refractory to AEDs, sadly we lost this patient when he was 2 years and six months old due to probable SUDEP, when no severe respiratory or cardiovascular disorders could be linked to his death. Prior to his death, his DQ indicated severe ID, which firmly associated *ARX* gene variants and severe ID/DD^35^. The linkage between *ARX* gene variants and SUDEP was previously considered as a potential cause of SUDEP^36^, but further studies are needed to emphasize this hypothesis, which firmly associated *ARX* gene variants and severe ID^35^, variants in this gene have been associated with X-linked severe ID, lissencephaly with abnormal genitalia^36^,^37^,^38^,^39^,^40^,^41^. Previous case control studies have suggested that epilepsy onset, AED polytherapy and poor seizure control are major risk factors for SUDEP^42^.

*De novo* variants in *CDKL5* are a well-recognized cause of EIEE and severe, Rett-like developmental delay^43^,^44^, we identified two *de novo CDKL5* gene variants in two case (S553 female, C0106 male) NM_003159, c.1110delC/p.N370fs and NM_003159 c.278dupA/p.E93fs respectively, now at the age of one year and five months (S553), is not able to sit independently, she cannot talk and sporadically shows random involuntary movements, (C0106) is two years and eight months old and cannot talk or walk, they both have severe ID, and their seizures are resistant to AEDs and/or ketogenic diet. *CDKL5* gene is located on the X chromosome, and the majority of reports describe *de novo* X-linked variants in females^43^,^45^, here, we identified a variant in a male patient, which spots the lights on the potential under-recognition of *CDKL5* gene mutation as a pathogenic variant in males, which has been recently emphasized^28^,^30^. Previously reported studies have shown that patients with *CDKL5* variants are mainly presented as early onset epileptic encephalopathy (EOEE) with epileptic spasms and severe ID, and suggested that *CDKL5* variants should be kept in consideration first in patients showing EOEE with involuntary movements which is being advised by our results which is being enforced by our results^28^,^30^,^46^.

In variants carrying cases, severe ID was found in 31% (4/13), moderate ID in 54% (7/13) and mild ID was found in 15% (2/13), this result demonstrates the well-established relation between unexplained EIEE and intellectual disabilities and enriches their genotype-phenotype correlations.

### Comparison with Other Cohort Studies of EE Tested by NGS

Zhang Y *et al*.^17^, used targeted next-generation sequencing to detect variants within 300 genes related to epilepsy and ID/DD in 253 Chinese children with unexplained epilepsy and ID/DD. The detection rate was 18% (46/253) in the whole group and 26% (17/65) in the early-onset (before three months after birth) epilepsy group, in their cohort, patients with an *SCN1A* variants accounted for the largest proportion, 17% (8/46), which matches our results when we found that *SCN1A* was the most frequently mutated gene in our study, accounting for 5 (38%) of 13 variants, emphasizing on the linkage between *SCN1A* variants and EIEE. Gokben S *et al*.^47^, reported a cohort of 30 patients of early-onset EE and identified twelve definite or potential causal variants using targeted next generation sequencing analysis. The detection rate in our study was 19% while in their study was 40%; this inconsistency could be due to that parental consanguinity was found in 40%of the cases and perinatal asphyxia was reported in 27% of the patients. Thus different inclusion criteria may account for inconsistent rate between similar studies. Recently, Zhang Q *et al*.^28^, reported a cohort of 175 Chinese patients with EOEEs, the author identified variants is 56 patients *de novo* heterozygous variants, unlike our study *CDKL5* gene mutation accounted for the largest proportion 13.1% (23/175). In their study, the majority of cases (68%, 119/175) remained unexplained which we found quite similar to our findings that (81%, 55/68) of our cases continued to be unexplained, this observation spots the light on the additional candidate pathogenic genes that still need to be unraveled in the future, *ARX* was a candidate gene in their cohort of 175 patients, but no detected variant was found in this gene, unlike our study. The author recommended VPA, LEV and TPM for patients with *KCNQ2* variants, which matches our observation, as VPA and LEV were effective in our patients with *KCNQ2* variants. Kong *et al*.^23^, reported a cohort of Chinese patients and identified five *de novo SCN8A* variants that stated to be the first reported in Chinese patients with epilepsy and ID/DD, in our cohort we identified a novel *de novo SCN8A* mutation *associated* with unclassified EEEs and moderate ID, thus enriching the relation between *SCN8A* variants and EIEE. Mercimek-Mahmutoglu *et al*.^48^, conducted a retrospective cohort study of 110 patients with intractable epilepsy, global developmental delay, and cognitive dysfunction, Detection rate by targeted next-generation sequencing was 12.7% and *SCN1A* was the most frequently mutated gene accounting for 29% of 14 variants, which we found similar to our results. Hardies k *et al*.^49^, reviewed 35 NGS studies that focused on patients with epilepsy, and cited that genetic factors are thought to have a role in 70% of all epilepsy; also the author reported that NGS findings have additionally increased the recognition of phenotypical and genetic heterogeneity which was demonstrated in our study.

### Summary

Through next-generation sequencing in 68 Han Chinese patients with unexplained EIEEs we were able to detect pathogenic variants and likely pathogenic variants in 15% and 4% of the cases, respectively. Six of these variants are novel. A total detection rate of 19% of variants adds weight to the known efficacy of next-generation sequencing in detecting variants in patients with unexplained EIEE. Moderate to severe ID were presented in eleven patients of the thirteen variants-carrying patients which augments the recognized relation between EIEE and moderate to severe ID, especially in patients with seizures onset within the first year of life. We advise that further attention should be paid to EIEEs patients with *ARX* gene variants, especially those who are on AED poly therapy and with poor seizure control, as we lost one patient with a variant in *ARX* gene due to SUDEP.

Our study not only helped to improve our understanding of the clinical characteristics and the possible etiology of EIEEs, but also enriched the EIEEs genes bank and enlightened our comprehension of EIEE-related concerns and would potentially serve as a valuable reference for further studies.

### Study Limitations

Parental testing was not available in three cases. Without results of segregation studies and *in vitro*/*in vivo* analyses, a low probability of pathogenicity should be considered, and translation into clinical practice should be implemented with caution. The difference in socio-cultural backgrounds and the small size of our cohort (68 patients) may have resulted in a different percentage of variants with mild and moderate ID compared to some previously reported studies.

## Additional Information

**How to cite this article**: Arafat, A. *et al*. Unexplained Early Infantile Epileptic Encephalopathy in Han Chinese Children: Next-Generation Sequencing and Phenotype Enriching. *Sci. Rep.* **7**, 46227; doi: 10.1038/srep46227 (2017).

**Publisher's note:** Springer Nature remains neutral with regard to jurisdictional claims in published maps and institutional affiliations.

## Supplementary Material

Supplementary Table S1

## Figures and Tables

**Table 1:** Summary of the Clinical Features of Patients.

Clinical Characteristics	*N*
Age of Seizure Onset	
<3 month	24
3~6 month	29
7~12 month	15
Sex	
Male:female	47:21
Family History	
Positive family history*	9
Mother’s Pregnancy History	
Spontaneous abortion	3
Threatened abortion	3
Pregnancy hypertension	1
Premature labour	2
EIEE Classification	
West syndrome	44
Dravet’s syndrome	4
Ohtahara syndrome	2
uEEEs	18
Seizure Types	
Spasms	33
Spasms-tonic	4
Tonic	3
Tonic clonic	12
Partial	9
Multiple seizure types	7
Head MRI or CT Scan	
Normal	39
Ventriculomegaly	18
Arachnoid cyst	3
Myelination delay	1
Formation of CSP	1
Cases where MRI or CT was not checked	6
EEG	
Hypsarrhythmia	48
Sharp waves	3
Slow waves	2
Slow-spike-and-wave complexes	2
Slow basic background activity	1
Burst-suppresion	4
Multiple epileptiform discharges	8
Intellectual Disabilities (ID)	
Mild	18
Moderate	20
Severe	30
Response to AEDs Therapy	
Seizures-free cases	13
Seizures-controlled cases^**^	16
AEDs resistant cases^***^	18
Deceased cases	3
Cases we lost contact with	8

*First-degree relative had history of epilepsy or intellectual disability.

**Seizures were considered controlled if the reduction of frequency of seizures attacks became more than 50% after treatment.

***Patients were considered resistant to treatment if the reduction of seizures attacks became less than 50% after taking AEDs.

**Table 2:** Summary of the 13 Cases with Variants Detected in Our Study.

Patient	Age of Onset	Clinical Diagnosis	Seizures types	EEG (age)	Neuroimaging	Response to AEDs	DQ/IQ results & Age at evaluation	ID	Mutant Gene, Inheritance	HGMD Reported or Novel	SIFT/Polyphen 2 Prediction	Mutation	Location of Variant (Protein)
	**Pathogenic Variants**												
C0106	1 month (M)	WS	Partial then epileptic spasms	Hypsarrhythmia	Ventriculomegaly	Resistant to AEDs and ketogenic diet	(30) 2 y and 8 mo	Severe	*CDKL5* Xp22.13, *de novo*	Novel	/	(NM_003159) chrX :18593605–18593606(insA) c.278dupA/p.E93fs	Protein kinase domain
S553	3 months (F)	WS	Partial then epileptic spasms	Hypsarrhythmia	Normal	Resistant to AEDs and ketogenic diet	(27) 1 y and 5 mo	Severe	*CDKL5* Xp22.13, *de novo*	(Zhao 2014)^1^	/	(NM_003159) chrX :18622154–18622155(delC) c.1110delC/p.N370fs	Cytoplasmic domain
S559	2 days (F)	uEEEs	Tonic	Sharp waves	Arachnoid cyst	PB then lev and VPA (not controlled) then monotherapy LEV (seizure-free)	(60) 4mo & (55) 4 y	Mild	*KCNQ2* 20q13.33, *de novo*	(Moulard 2001)^2,****^	Deleterious/probably damaging	(NM_004518) chr20:62044908(G > A) c.1574G > A/p.R525Q	C-terminal
C0107	1 months (F)	uEEEs	Partial	Spikes, slow-spike-and-wave and polyspike-and-wave complexes	Normal	LEV then LEV and VPA (seizures-controlled)	(42) 1 y and 6 mo	Moderate	*KCNQ2* 20q13.33, NT	(Moulard 2001)^2,****^	Deleterious/probably damaging	(NM_004518) chr20:62044908(G > A) c.1574G > A/p.R525Q	C-terminal
S557	4 months (M)	uEEEs	Tonic	Hypsarrhythmia, Spike wave (11 months)	Ventriculomegaly	LEV then LEVPB and CBZ, then VPA (refractory seizures)	(36) 2 y	Moderate	*SCN8A* 12q13.13, *de novo*	Novel	Deleterious/probably damaging	(NM_014191) chr12:52200885(G > A) c.5615G > A/p.R1872Q	Calmodulin-binding motif
C0125	6 months (F))	DS	Partial, myoclonic Then tonic	Normal, then spikes,sharp waves and polyspikes (7 months) Slow wave and abnormal background (4 years)	Formation of CSP	OXC, LEV, TPM, then ketogenic diet (slightly-controlled)	(40) 4 y and 2 mo	Moderate	*SCN1A* 2q24.3*, de novo*	(Sugawara 2002)^3^	Tolerated/probably damaging	(NM_001202435) chr2:166898844(C > T) c.2134C > T/p.R712X	Cytoplasmic domain
C0129	7 months (M)	DS	Tonic clonic, Febrile convulsions (13 months), tonic, tonic clonic and partial (2y 7months)	Slow-spike-and- wave complexes	Normal	VPA controlled for 1 year, then LEV (controlled, but induced by fever)	(55) 3 y & (52) 5 y	Mild	*SCN1A* 2q24.3, *de novo*	Novel	Tolerated/probably damaging	(NM_001202435) chr2:166929907(G > T) c.225G > T/p.E75D	Cytoplasmic domain
R1014	6 months (M)	DS	Fever-induced tonic or tonic clonic	Normal then spikes, sharp waves and polyspikes (10 months)	Normal	PB, LEV and TPM (slightly-controlled)	(38) 2 y and 6 mo	Moderate	*SCN1A* 2q24.3, *de novo*	Novel	Deleterious/probably damaging	(NM_001202435) chr2:166850697(G > A) c.4811G > A/p.W1604X	Ion transport domain
C0108	3 months (M)	WS	spasms	Hypsarrhythmia	Arachnoid cyst	ACTH,TPM and VPA (refractory seizures) Then TPM, VPA and ketogenic diet (refractory seizures)	(19) 3 y and 10 mo	Severe	*STXBP1* 9q34.11, *de novo*	(Allen 2016)^4^	Deleterious/probably damaging	(NM_003165) chr9:130438188(C > T) c.1216C > T/p.R406C	Syntaxin-binding protein 1 chain
R1007	3 months (M)	OS	Spasms	Intermittent burst-suppression during sleep cycle	Ventriculomegaly	VPA and ACTH (refractory seizures)	(36) 1 y	Moderate	*STXBP1* 9q34.11*, de novo*	(Allen 2016)^4^	Deleterious/probably damaging	(NM_003165) chr9:130438188(C > T) c.1216 C > T/p.R406C	Syntaxin-binding protein 1 chain
	**Likely Pathogenic Variants**												
C0117	7 months (M)	uEEEs	Partial	Slow waves with high amplitude	Ventriculomegaly	VPA, TPM and LEV (refractory seizures)	(44) 1 y and 6 mo	Moderate	*SCN1A* 2q24.3, NT	Novel	Deleterious/probably damaging	(NM_001202435) chr2:166859090(T > A) c.4176T > A/p.N1392K	Ion transport domain
S560	1 year (F)	uEEEs	Tonic	Slow-spike-and-wave complexes 100% during NREM sleep cycle	Myelination delay in the cerebral white matter	VPA then LEV and VPA (refractory seizures)	(37) 2 y and 4 mo	Moderate	*SCN1A* 2q24.3, NT	***	Deleterious/probably damaging	(NM_001202435) chr2:166900519(G > A) c.1703G > A/p.R568Q	Cytoplasmic domain
S569	53 days (M)	OS developed to WS	Spasms	Intermittent burst-suppression during sleep cycle	Normal	ACTH and LEV (seizure controlled) then refractory seizures after 4 months. Then LEV and VPA (refractory seizures) SUDEP	(21) 2 y and 6 mo	Severe	*ARX* Xp21.3, maternal	Novel	Deleterious/probably damaging	(NM_139058) chrX:25022876 (G > C) c.1600G > C/p.A534P	Aristaless domain

(M) Male; (F) Female; (OS) Ohtarah syndrome; (WS) West syndrome; (DS) Dravet syndrome; (uEEEs) unknown type of early epileptic encephalopathy; (NT) not tested; (SUDEP) sudden unexpected death in epilepsy; (y) year; (mo) month; (ACTH) adrenocorticotropic hormone; (OXC) Oxcarbazepine; (CBZ) Carbamazepine; (LEV) Levetiracetam; (PB) Phenobarbital; (TPM) Topiramate (VPA); Sodium valproate (VPA); (NREM) non-rapid eye movement.

^1^(Zhao 2014): The variant was reported in variants of *CDKL5* and early-onset epileptic encephalopathies or Hanefeld variants of RTT(Rett syndrome); Zhao, Y. *et al*. Clinical features and gene mutational spectrum of *CDKL5*-related diseases in a cohort of Chinese patients. *BMC Med. Genet.* **25**, 15:24 (2014)^50^.

^2^(Moulard 2001): The variant was reported in the association between the benign neonatal epilepsy and variants in genes coding for potassium channel subunit *KCNQ*2; Moulard, B. *et al*. Ion channel variation causes epilepsies. *Brain Res. Brain Res. Rev.* **36**, 275–284 (2001)^51^.

^3^(Sugawara 2002) : The variant was reported in Myoclonic epilepsy of infancy; Sugawara, T. *et al*. Frequent variants of SCN1A in severe myoclonic epilepsy in infancy. *Neurology* **58**, 1122–1124 (2002)^16^.

^4^(Allen 2016): The variant was reported in Unexplained early onset epileptic encephalopathy; Allen, N. M. *et al*. Unexplained early onset epileptic encephalopathy: Exome screening and phenotype expansion. Epilepsia **57**, e12–7 (2016)^31^.

***The variant is not reported in HGMD and the ExAC_MAF = 0, but it is reported in dbSNP build 146 rs794727025 dbSNP: rs794727025 Position: chr2:166900519 Band: 2q24.3.

****The variant is not reported in the ExAC, but it is reported in dbSNP build 146 rs118192234 dbSNP: rs118192234 Position: chr20:62044908 Band: 20q13.33.

*****Although parental testing was not available, but since (S559 and C0107) share the same variant (c.1574G > A; p.R525Q) in *KCNQ2*, since this variant is pathogenic in patient S559, the same variant of patient C0107 was considered pathogenic according to the ACMG Standards and guidelines for the interpretation of sequence variants^12^.
