# Supplementary material for: Unexplained Early Infantile Epileptic Encephalopathy in Han Chinese Children: Next-Generation Sequencing and Phenotype Enriching
Source: Sci Rep. 2017 Apr 7;7:46227. doi: 10.1038/srep46227 (PMC5384237; doi:10.1038/srep46227)
Supplement: Supplementary Table S1 [file srep46227-s1.pdf]

Unexplained Early Infantile Epileptic Encephalopathy in Han Chinese Children: Next-Generation Sequencing and Phenotype Enriching

Ahmed Arafat[1], Peng Jing[1], Yuping Ma[1], Miao Pu[1], Gai Nan[1], He Fang[1], Chen Chen[1], Yin Fei[1] *

1. Xiang Ya First Hospital of Central South University, China
87 Xiang ya road, Changsha, Hunan, P.R. China, 410008
Tel: 86-731-84327330
Fax: 86-731-84327332
E.mail: csxyhpo@163.com

*Corresponding Author

Supplementary documents:
Supporting Information

Table S1. A list of 308 candidate genes associated with Early Infantile Epileptic Encephalopathy (EIEE).

ABCC8	*KCNQ3*	*DMPK*	*PTPN22*	*C4A*	*NDUFS6*	*GABRG2*	*SLC46A1*
ACADSB	*KCTD7*	*DNAJC5*	*PUS1*	*CACNA1H*	*NDUFV1*	*GAMT*	*SLC9A6*
ACTB	*KDM5C*	*DNASE1*	*QDPR*	*CACNB4*	*NDUFV2*	*GBA*	*SLC9A9*
ACY1	*KIF11*	*DOCK6*	*RAB39B*	*CACNG2*	*NEU1*	*GCK*	*SNIP1*
ADK	*KIF1A*	*DPYD*	*RANBP2*	*CASR*	*NF1*	*GCSH*	*SNRPN*
ADSL	*KRAS*	*DRD2*	*RELN*	*CCM1*	*NHLRC1*	*GPHN*	*SOBP*
AFG3L2	*L2HGDH*	*DRD3*	*ROGDI*	***CDKL5***	*NHS*	*GLB1*	*SPAST*
AKT1	*LBR*	*DTNBP1*	*RPIA*	*CEP152*	*NOTCH3*	*GLDC*	***SPTAN1***
ALDH7A1	*LGI1*	*DXS423E*	*RTN4R*	*CHI3L1*	*NR3C1*	*GLRA1*	*SPTLC2*
ALG1	*LIAS*	*EBP*	*RYR1*	*CHRNA2*	*NRXN1*	*GOSR2*	*SRPX2*
ALG11	*LMX1B*	*ECM1*	*SCARB2*	*CHRNA3*	*NTNG1*	*GPR48*	*STRADA*
ALG3	*MAGI1*	*EFHC1*	***SCN1A***	*CHRNA4*	*NUBPL*	*GPR98*	*STS*
AMACR	*MAGI2*	*EHMT1*	***SCN1B***	*CHRNA5*	*OFD1*	*GRIN1*	***STXBP1***
AMT	*MAN1B1*	*EIF2B1*	***SCN2A***	*CHRNA7*	*OPHN1*	*GRIN2A*	*SUOX*
APOL2	*MANBA*	*EIF2B2*	***SCN8A***	*CHRNB2*	*PAFAH1B1*	*GSS*	*SYN1*
APOL4	*MAPK10*	*EIF2B3*	*SCN9A*	*CLCN2*	*PAH*	*GYS1*	*SYN2*
APP	*MCCC2*	*EIF2B4*	*SCZD1*	*CLN3*	*PAK3*	*HAX1*	***SYNGAP1***
ARG1	*MCPH1*	*EIF2B5*	*SCZD11*	*CLN5*	*PANK2*	*HFE*	*SYP*
ARHGAP31	*MECP2*	*ELP4*	*SCZD12*	*CLN6*	***PCDH19***	*HLA-DQA1*	***TBC1D24***
ARHGEF9	*MEF2C*	*EMX2*	*SCZD2*	*CLN8*	*PDHA1*	*HLA-DQB1*	*TBP*
ARSA	*MFSD8*	*EPB41L1*	*SCZD3*	*CNTNAP2*	*PGK1*	*HNF1B*	*TCF4*
ARSE	*MLC1*	*EPM2A*	*SCZD5*	*COG7*	*PHF6*	*HP*	*TMEM165*
ARX	*MOCS1*	*ERBB4*	*SCZD6*	*COH1*	*PHGDH*	*HRAS*	*TPP1*
ASAH1	*MOCS2*	*ERLIN2*	*SCZD7*	*COMT*	*PIGL*	*HSD17B10*	*TREM2*

ATIC	*MOCS3*	*EVC*	*SCZD8*	*COX6B1*	*PIGV*	*HSD17B4*	*TREX1*
ATN1	*MR1*	*FADD*	*SERPINI1*	*CPA6*	*PLA2G6*	*HTR2A*	*TSC1*
ATP1A2	*MTHFR*	*AMER1*	*SETBP1*	*CPS1*	***PLCB1***	*HTT*	*TSC2*
ATP2A2	*NDN*	*FASTKD2*	*SGCE*	*CSTB*	*PLP1*	*ICCA*	*TSEN2*
ATP6AP2	*NDUFA1*	*FCGR2B*	*SHH*	*CTSA*	***PNKP***	*IDH2*	*TSEN34*
ATRX	*NDUFA11*	*FKTN*	*SIAT9*	*CTSD*	*PNPO*	*IDS*	*TSEN54*
ATXN10	*NDUFAF1*	*FLNA*	*SIX3*	*CYB5R3*	*POLG*	*IER3IP1*	*TUBGCP6*
BANK1	*NDUFAF2*	*FOLR1*	*SLC17A5*	*D2HGDH*	*POMGNT1*	*IFNG*	*TYROBP*
BOLA3	*NDUFAF3*	*FOXG1*	*SLC19A3*	*DAO*	*PPOX*	*IL6*	*UBE3A*
BRP44L	*NDUFAF4*	*FOXRED1*	*SLC20A2*	*DAOA*	*PPT1*	*INS*	*XK*
C10orf2	*NDUFB3*	***KCNQ2***	*SLC25A15*	*DBH*	*PRICKLE1*	*KCNA1*	*ZDHHC15*
C12orf62	*NDUFS1*	***GABRA1***	***SLC25A22***	*DCX*	*PRICKLE2*	*KCNJ10*	*ZEB2*
C20orf7	*NDUFS2*	*GABRB3*	*SLC26A4*	*DHFR*	*PROC*	*KCNJ11*	*ZFYVE26*
C2orf64	*NDUFS4*	*GABRD*	*SLC2A1*	*DISC1*	*PRODH*	*KCNMA1*	*ZNF41*
				DISC2	*PRRT2*	*KCNQ1*	***KCNT1***

Table S1. A list of 308 candidate genes associated with (EIEE) *in bold are already known pathogenic genes.*
